# DNA methylation may affect beef tenderness through signal transduction in *Bos indicus*

**DOI:** 10.1186/s13072-022-00449-4

**Published:** 2022-05-13

**Authors:** Marcela Maria de Souza, Simone Cristina Méo Niciura, Marina Ibelli Pereira Rocha, Zhangyuan Pan, Huaijun Zhou, Jennifer Jessica Bruscadin, Wellison Jarles da Silva Diniz, Juliana Afonso, Priscila Silva Neubern de Oliveira, Gerson B. Mourão, Adhemar Zerlotini, Luiz Lehmann Coutinho, James E. Koltes, Luciana Correia de Almeida Regitano

**Affiliations:** 1 Empresa Brasileira de Pesquisa Agropecuária, Embrapa Pecuária Sudeste, São Carlos, Brazil; 2 Department of Animal Science, Iowa State University, Ames, USA; 3 Department of Genetics and Evolution, Federal University of São Carlos, São Carlos, Brazil; 4 Department of Animal Science, University of California, Davis, CA USA; 5 Department of Animal Science, Auburn University, Auburn, Alabama, USA; 6 Department of Animal Science, Luiz de Queiroz College of Agriculture, University of São Paulo, Piracicaba, Brazil; 7 Embrapa Informática Agropecuária, Empresa Brasileira de Pesquisa Agropecuária, Campinas, Brazil

**Keywords:** Nelore, RRBS, Cattle, *GNAS*, *EBF3*, Shear force, Epigenome, Muscle, Methylation

## Abstract

**Background:**

Beef tenderness is a complex trait of economic importance for the beef industry. Understanding the epigenetic mechanisms underlying this trait may help improve the accuracy of breeding programs. However, little is known about epigenetic effects on *Bos taurus* muscle and their implications in tenderness, and no studies have been conducted in *Bos indicus*.

**Results:**

Comparing methylation profile of *Bos indicus* skeletal muscle with contrasting beef tenderness at 14 days after slaughter, we identified differentially methylated cytosines and regions associated with this trait. Interestingly, muscle that became tender beef had higher levels of hypermethylation compared to the tough group. Enrichment analysis of predicted target genes suggested that differences in methylation between tender and tough beef may affect signal transduction pathways, among which G protein signaling was a key pathway. In addition, different methylation levels were found associated with expression levels of *GNAS, PDE4B*, *EPCAM* and *EBF3* genes. The differentially methylated elements correlated with *EBF3* and *GNAS* genes overlapped CpG islands and regulatory elements. *GNAS*, a complex imprinted gene, has a key role on G protein signaling pathways. Moreover, both G protein signaling pathway and the *EBF3* gene regulate muscle homeostasis, relaxation, and muscle cell-specificity.

**Conclusions:**

We present differentially methylated loci that may be of interest to decipher the epigenetic mechanisms affecting tenderness. Supported by the previous knowledge about regulatory elements and gene function, the methylation data suggests *EBF3* and *GNAS* as potential candidate genes and G protein signaling as potential candidate pathway associated with beef tenderness via methylation.

**Supplementary Information:**

The online version contains supplementary material available at 10.1186/s13072-022-00449-4.

## Background

Tenderness is one of the most appreciated beef quality traits by consumers [1], which makes this a desired trait by producers. Knowledge of the biological processes controlling this trait is crucial to improve beef quality through better management decisions and animal breeding. Beef tenderness is a complex trait with intra and inter-breed variability [2]. It is well known that most *Bos taurus* breeds present a more tender beef product when compared to beef sourced from *Bos indicus* [3]. Thus, the mechanisms impacting tenderness that differ between these different subspecies merit investigation. As Nelore is the predominant *Bos indicus* breed in Brazil, and Brazil is one of the biggest exporters of bovine beef in the world [4], knowledge of specific mechanisms affecting tenderness in *Bos indicus* may have great impact in the international beef market.

Omics studies exploring the biological mechanisms underlying tenderness in beef cattle have been developed for both *Bos taurus* [5] and *Bos indicus* [6–11]. However, the knowledge regarding the role of epigenetics on tenderness is still limited, mainly for *Bos indicus*.

Epigenetics refers to modifications that occur in gene expression without altering the DNA sequence, i.e., modifications that alter the phenotype without modifying the genotype [12, 13]. Methylation is one of the most common and more studied epigenetic marks in mammals and consists of the addition of a methyl group in a DNA cytosine [12, 14–16]. In general, there is an inverse correlation between DNA methylation (DNAm) and gene expression level [17]. Higher levels of methylation in the promoter region are related to gene silencing [14], whereas promoters of active genes show lower intensity of methylation [15]. The effect of CpGs methylation on gene expression is modulated by complex mechanisms and depends on its genomic location, being context-dependent [18]. Studying human cells, Varley and colleagues [18] found that methylation of CpGs close to transcription start site (TSS) generally has repressing effect on gene expression; however, methylated CpGs in gene body and far from TSS may have either positive or negative correlation with expression, depending on its genome context. For instance, if the gene body methylated CpG is in CpG islands (CpGi), either positive or negative correlation was found, while those not located in CpGi were positively correlated with gene expression.

Few studies in bovine muscle have investigated the methylation profile, as reviewed by Wang and Ibeagha-Awemu [19], and there is only one relating it to beef tenderness [20]. Among these few studies, Huang et al. [21] compared the methylation profile and its relationship with mRNA and miRNA expression levels in fetuses and adult muscle of Chinese Qinchuan cattle. Lately, the methylome of muscle was studied in Angus [20] and in a crossed *taurus vs. indicus* Brangus-Angus population [22]. The unique study analyzing muscle methylation and tenderness was recently published by Zhao et al. [20] that identified several differentially methylated regions between animals with higher and lower tenderness in the *Bos taurus* population. To our knowledge, there are no studies available investigating the relationship between methylation and tenderness in *Bos indicus* animals. Therefore, since *Bos taurus* and *Bos indicus* may present difference in their phenotype and transcriptomes, we also expect differences in methylation between them, thus turning methylation studies in *Bos indicus* a need for the comprehension of molecular basis guiding tenderness. The objective of this study was to investigate the association of DNA methylation profile in Nelore (*Bos indicus*) skeletal muscle by comparing contrasting animals for tenderness. In addition, to find methylation sites that are candidates to affect the trait, we overlaid our results with previous QTL studies in the same population. Furthermore, we screened whether these regions are in known regulatory elements using the recently published database from the FAANG pilot project [23] and verified the relationship between methylation and gene expression in muscle.

## Results

### Animals and phenotypic data

In the present work we used two groups of animals subsampled from a 200 population to represent the extremes of the shear force (SF) distribution, a measure of beef tenderness. The group of animals showing the lowest tenderness (Tough group, *n* = 6), had higher estimated breeding value for shear force (EBVSF), with average ± standard error of 0.64 ± 0.058. The group of animals presenting higher beef tenderness had lower EBVSF values (Tender group, *n* = 6), with average ± standard error of − 0.82 ± 0.099. Groups' sample composition, phenotypes and EBVSF values are presented in Additional File 2: Table S1. The averages of SF measurements and EBVSFs were different between the two groups with a *t*-Student *p* = 1.227e−06 and *p* = 1.483e−07, respectively (Additional file 1: Fig. S1).

### Reduced representation bisulfite sequencing (RRBS) and Nelore muscle methylation profile

We performed RRBS in both tough (*n* = 6) and tender (*n* = 6) muscle samples to access the DNA methylation status and to identify differentially methylated cytosines (DMCs) and regions (DMRs) between the two tenderness phenotypes. The sequence depth ranged from 29 to 55 million reads per sample with an average of 20 million reads per sample aligned to the ARS-UCD1.2/bosTau9 genome and average mapping efficiency of 47.34%. A full summary of read mapping statistics is presented in Additional file 2: Table S2. Approximately, 275 million cytosines were analyzed, ranging from 203 to 353 million per sample. We observed that an average of 23.02% of all the analyzed cytosines (including non-CpGs) captured in the RRBS were cytosines followed by guanine, i.e., CpG, and 9.03% were methylated CpGs (Additional file 2: Table S3). From these CpGs, 635,469 were identified in the 12 samples and were used for the differential methylation analysis. Most of these CpGs overlapped intergenic regions (54%), followed by promoters (29%) and introns (11%), with exons overlapping the lowest number of CpGs analyzed (6%) (Additional file 1: Figure S2A). Around 60% of the cytosines analyzed were in CpG islands, while those overlapping CpG shores represented only 15% (Additional file 1: Figure S2B). We tested 635,469 CpGs and 193,249 regions that were common in the 12 samples. We found a low variability in global methylation profile across samples, as shown by the remarkably similar correlations, which ranged from 0.94 to 0.96 (Additional file 1: Figure S3).

### Most of the differentially methylated CpG and regions were hypermethylated in animals with higher tenderness

Although we observed a low global variation in the methylation profile, we identified 123 DMCs and 42 DMRs showing methylation differences between the two groups of extreme EBVSFs for SF at 14 days of aging (*q* value < 0.05 and methylation difference > 25%). Interestingly, most of the DMCs and DMRs had higher levels of methylation in the tender group (*n* = 87 and *n* = 36, respectively) than in the tough (*n* = 37 and *n* = 6, respectively). All the DMCs and DMRs are described in Additional file 2: Tables S4 and S5, respectively.

### Genome distribution of differentially methylated cytosines and regions

**Fig. 1:**
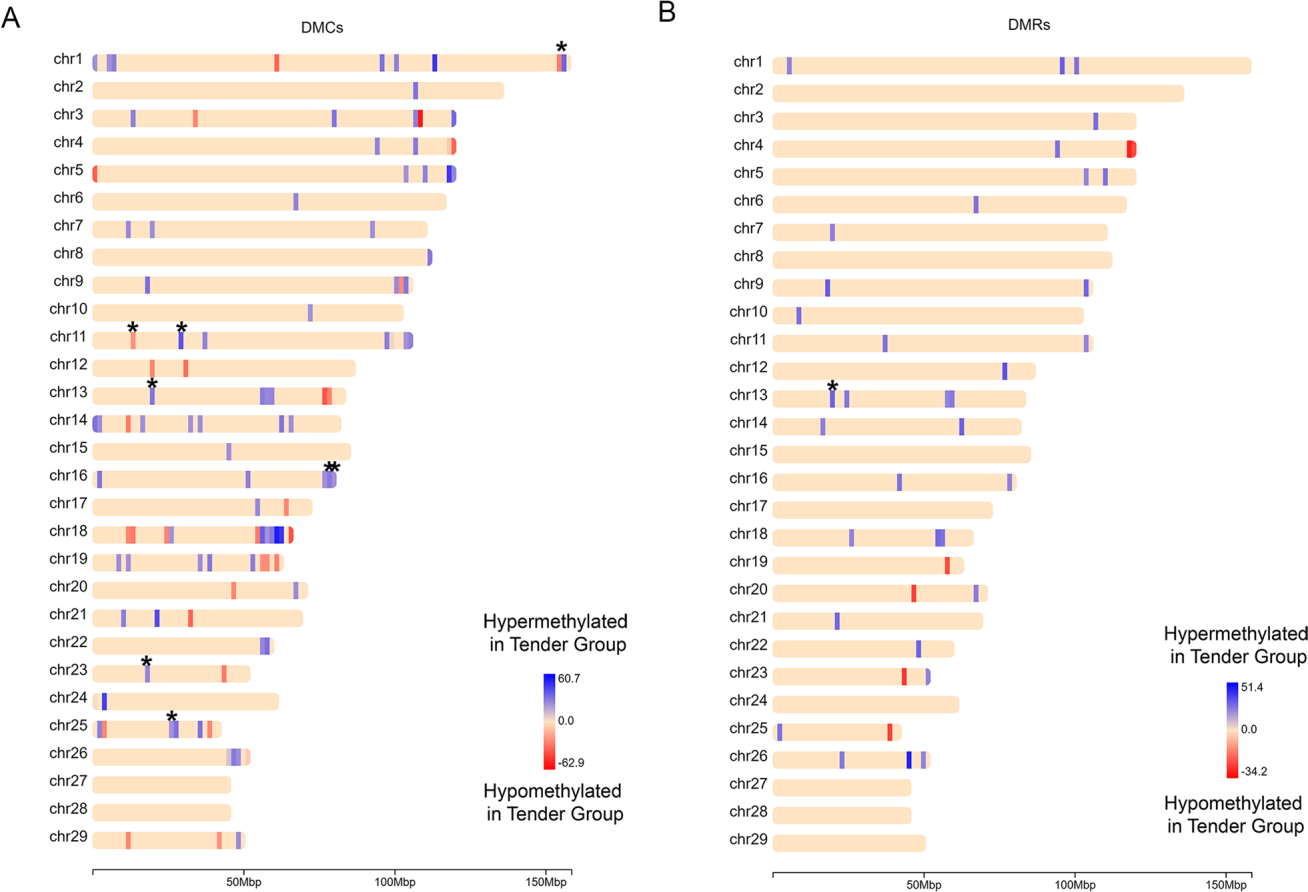
Chromosome distribution of **A** 123 differentially methylated cytosines (DMCs) and **B** 42 differentially methylated regions (DMRs) between two Nelore groups of divergent phenotypes for shear force at 14 days of aging. Only DMCs and DMRs showing *q* value < 0.05 and differences in methylation > 25% between groups are shown. The asterisks at the top of the represented DMCs and DMRs show which methylation feature overlapped with tenderness-related QTLs found in our population or QTL database. Blue bars represent DMCs or DMRs that were hypermethylated in animals with tender beef, while red bars represent those hypomethylated in the same group. The color gradient represents the difference of methylation (%) between the divergent groups

Thirty-two DMRs (76.19%) contained at least one DMC. We found DMCs in almost all chromosomes, except for BTA27 and BTA28, while DMRs were found on 21 different chromosomes (Fig. 1). Regarding the functional location, DMCs had a higher frequency in intergenic regions (66.67%) followed by the gene body: intron (21.74%), exon (9.76%) and promoters (2.44%) (Additional file 1: Figure S4A). On the other hand, DMRs were more present in introns (45.25%), followed by intergenic regions (35.71%), promoters (11.9%) and exons (7.14%) (Additional file 1: Figure S4B). As Additional file 1: Figure S5 shows, some DMCs and DMRs were in more than one functional location. Thus, only one functional location was considered for each DMC and DMR in calculating the percentage of the DMCs and DMRs located in promoters, exons, introns and intergenic regions, with preference in the following order promoter > exon > intron, as described by Methylkit version 1.8.1 [24]. In addition, most of the DMCs and DMRs were found outside of CpG island and CpG shore regions (Additional file 1: Figure S4C and D, respectively).

### Enrichment analysis of target genes suggests different methylation levels controlling genes acting on signal transduction

**Fig. 2:**
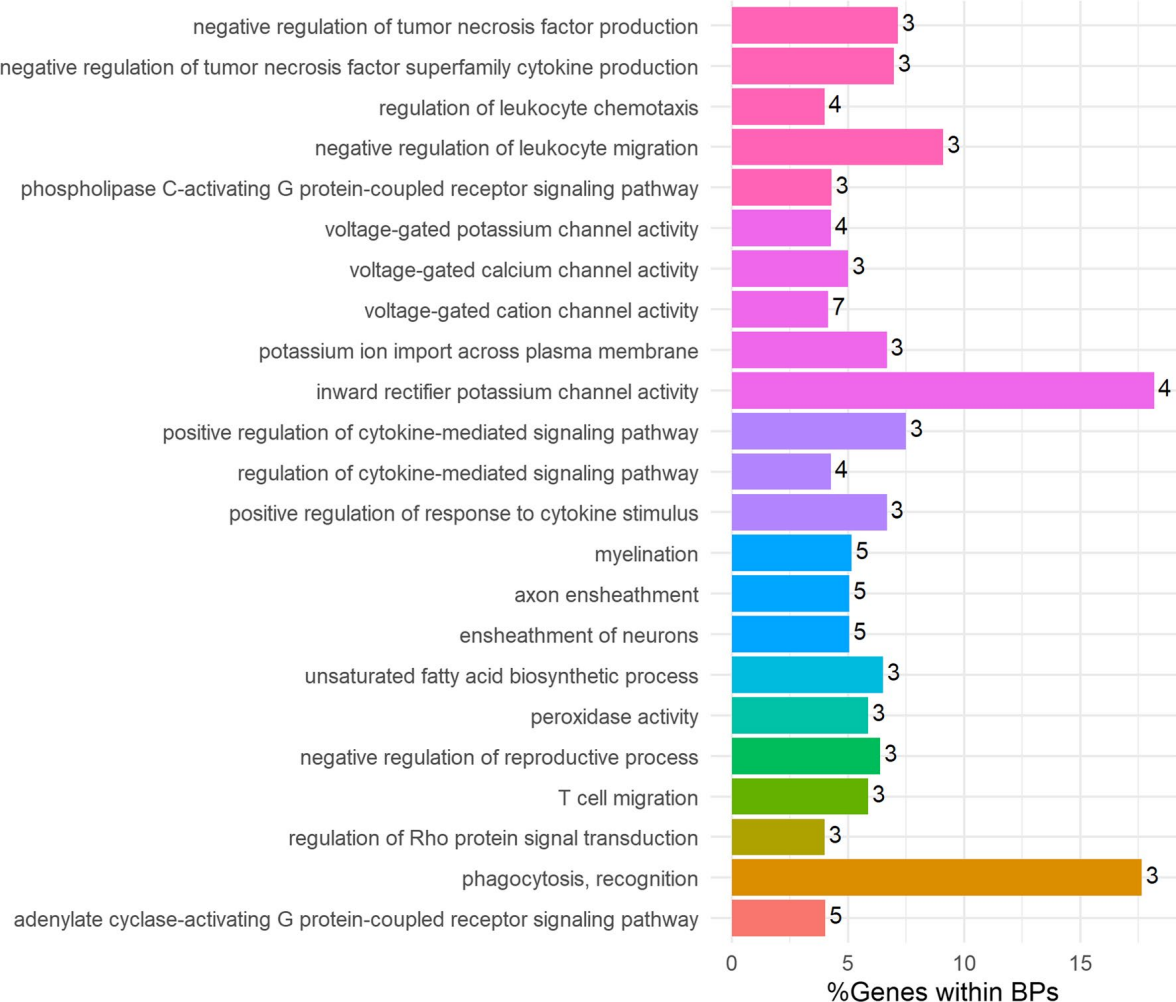
Overview of the over-represented biological processes (BP) annotations for the predicted target genes of differentially methylated CpGs (DMCs) and regions (DMRs). The bar plot displays result from a functional enrichment analysis to identify BP gene ontology (GO) terms. Significant ontology terms were identified at an estimated false discovery rate (FDR) < 0.05. The colored bars represent the percentage of genes per GO BP term and the number of genes associated with the term is shown as a label. Ontology terms that were closely related based on similar genes/biological functions were grouped by kappa score (kappa score > 0.4) and represented within the figure as the same color

Based on the annotations generated by the FAANG Consortium [23], enriched target genes for DMCs and DMRs were identified and are available in Additional file 2: Table S6. In total, 23 biological processes (BPs) gene ontology (GO) terms were enriched and clustered in 11 groups using the kappa score. Several signal transduction pathways were enriched (Fig. 2) in four different groups represented by positive regulation of cytokine-mediated signaling pathway (GO:0,001,961), regulation of Rho protein signal transduction (GO:0,035,023), phospholipase C-activating G protein-coupled receptor signaling pathway (GO:0,007,200) and adenylate cyclase-activating G protein-coupled receptor signaling pathway (GO:0,007,189). One group represented by inward rectifier potassium channel activity clustered five biological processes related to ions channel activity (GO:0,005,242). We also found three biological processes related to neurons grouped and represented by myelination (GO:0,042,552). Other five group had a unique biological process. Phagocytosis and inward rectifier potassium channel activity had around 18% of its associated genes enriched in our analysis, while the other pathways ranged between 4 and 9%. A complete description of ClueGo results, containing all gene ontologies, statistics, and genes clustered is available in Additional file 2: Table S7.

### DMCs and DMRs overlapped QTLs for shear force

To find DMCs and DMRs that may be also in QTLs related with tenderness, we looked for overlapping shear force (SF) QTLs identified in a previous *Bos indicus* study [6]. Six DMCs overlapped QTLs associated with shear force at day 14 after slaughter, from which two also overlapped QTLs for SF at 24 h after slaughter. All but one of these DMCs were hypermethylated in the tender muscle. Moreover, we found DMC8 and DMC83 overlapping QTLs for shear force from studies in other populations. Only one DMR (DMR18) overlapped a QTL, which was associated with shear force at day 14. Additional file 2: Tables S4 and S5 describe the QTLs overlapping DMCs and DMRs, respectively.

### Manually curated list of candidate methylation regions and cytosines affecting tenderness

The initial genome-wide DNAm screen, the results from enrichment analysis and the available information in the literature provided information for the development of a manually selected gene list (Additional file 2: Table S8). Thirty-two DMCs and nine DMRs were potentially acting as regulators of tenderness and differences in methylation level may result in phenotypic changes. Eight DMCs and one DMR overlapped QTLs for shear force and had 15 targets or closest genes with expression data. The enrichment analysis resulted in 27 genes that had expression data and were targets of 22 DMCs and 8 DMRs. Then considering the target genes or closest genes, we manually checked for function descriptions available in the literature related to muscle development or tenderness. This resulted in the addition of myosin VI (*MYO6*) and EBF transcription factor 3 (*EBF3*) genes.

### Four genes had expression correlated with methylation

**Fig. 3:**
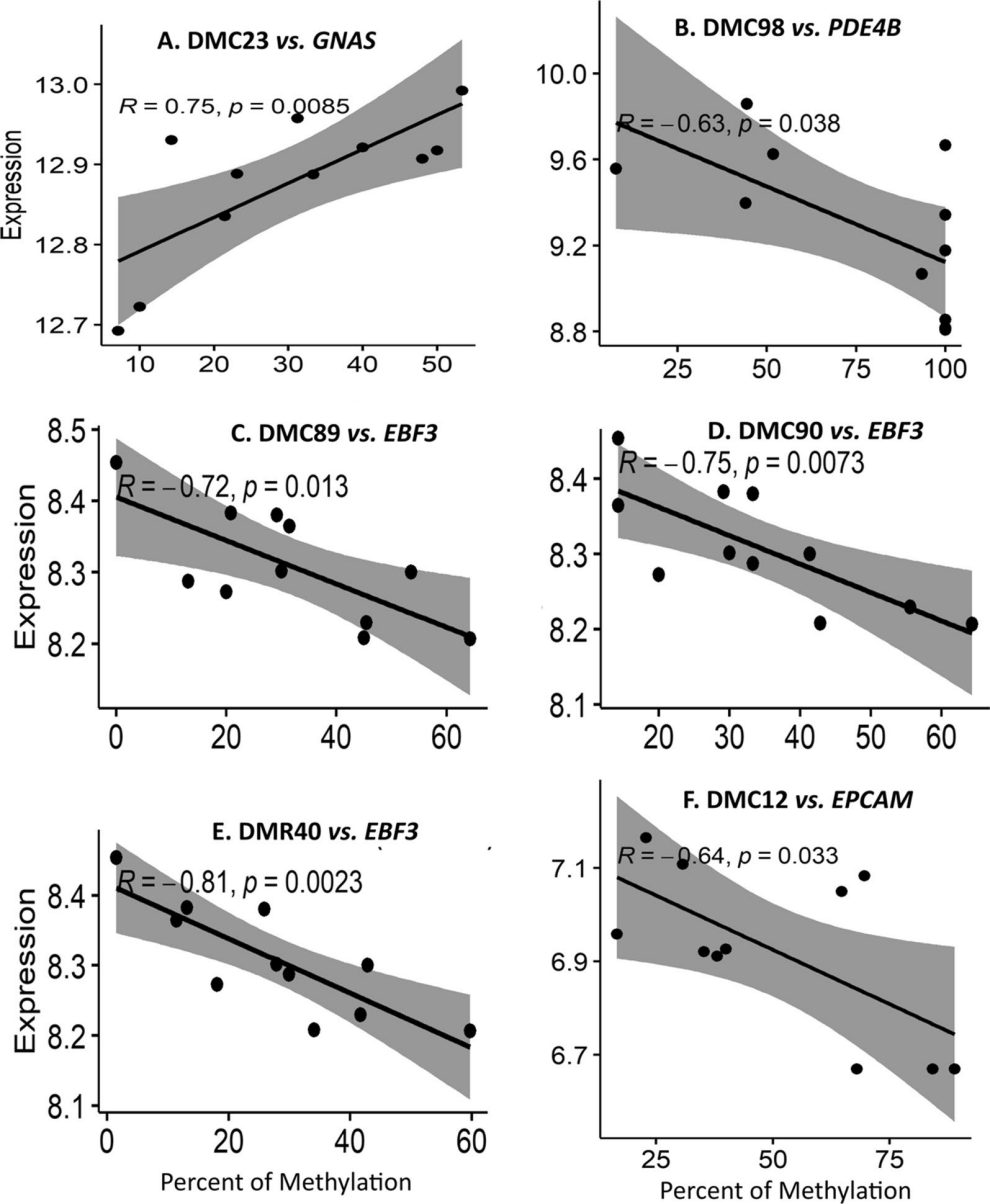
Significant Pearson coefficient correlation between the methylation percentage of DMCs and DMRs with the expression of its target genes (*p* < 0.05), using 11 RNA-Seq Nelore muscle samples. **A** Percent of methylation of DMC23 was positively correlated with the expression of gene *GNAS*. **B** Percent of methylation of DMC98 was negatively correlated with the expression of gene *PDE4B.* Percent of methylation of DMC89 (**C**), DMC90 (**D**) and DMR40 (**E**) were negatively correlated with expression of *EBF3* gene. **F** Percent of methylation of DMC12 was negatively correlated with the expression of gene *EPCAM*

We performed statistical association tests between the methylation percent of the selected DMCs/DMRs and the expression of its target genes. Eleven animals had paired RRBS methylation and RNA-Seq expression data. The RNA-Seq data showed an average of 9 billion reads with around 95% being uniquely mapped. RNA-Seq alignment results are described in Additional file 2: Table S9. We tested 65 combinations of DMC/DMRs and genes, which were 32 DMCs with 41 genes and 9 DMRs with 11 genes (Additional file 2: Table S8). DMC12, DMC 23, DMC89, DMC90, DMC98 and DMR40 showed a high correlation (*p* < 0.05) with one of their predicted target or closest genes, as shown in Table 1 and Fig. 3. The four genes were epithelial cell adhesion molecule (*EPCAM*), phosphodiesterase 4B (*PDE4B*), GNAS complex locus (*GNAS*) and *EBF3.* Besides the DMC12 and DMC98, the other three DMCs and DMR40 were in CpG islands and regulatory elements (Additional file 2: Table S10). In addition, we tested the differential expression between extremes for these four genes and we found *PDE4B* with higher expression in Tough (*p* = 0.017), while *GNAS* was higher in Tender group (*p* = 0.030) (Additional file 1: Figure S6). *EPCAM* and *EBF3* did not show differential expression (*p* > 0.05) between animals with divergent tenderness values.

**Table 1:** DMCs and DMRs that presented high correlation (Pearson; *p*-value < 0.05) between methylation level and gene expression of their predicted targets

DM	CpGi	Regulatory element state		Target gene^§^	*r*	*p*
		Muscle	Adipose			
DMC12	Other	ATAC island	Medium enhancer with ATAC	*EPCAM*	− 0.64	0.033
DMC98	Other	Quiescent	Quiescent	*PDE4B*	− 0.63	0.038
DMC23	CpGi	Bivalen/poised TSS	Strongly active promoter/transcript	*GNAS*	0.75	0.0085
DMC89	CpGi	strongly active promoters/transcripts	Bivalent/poised TSS	*EBF3*	− 0.72	0.013
DMC90	CpGi	strongly active promoters/transcripts	Bivalent/poised TSS	*EBF3*	− 0.75	0.0073
DMR40	CpGi	strongly active promoters/transcripts	Bivalent/poised TSS	*EBF3*	− 0.81	0.0023

The table includes whether the DMC or DMR overlaps a CpG island (CpGi), the regulatory element overlapped by the DMC or DMR in muscle and adipose tissues of *Bos taurus*, the target gene and the statistical association analysis results

#### *GNAS*

**Fig. 4:**
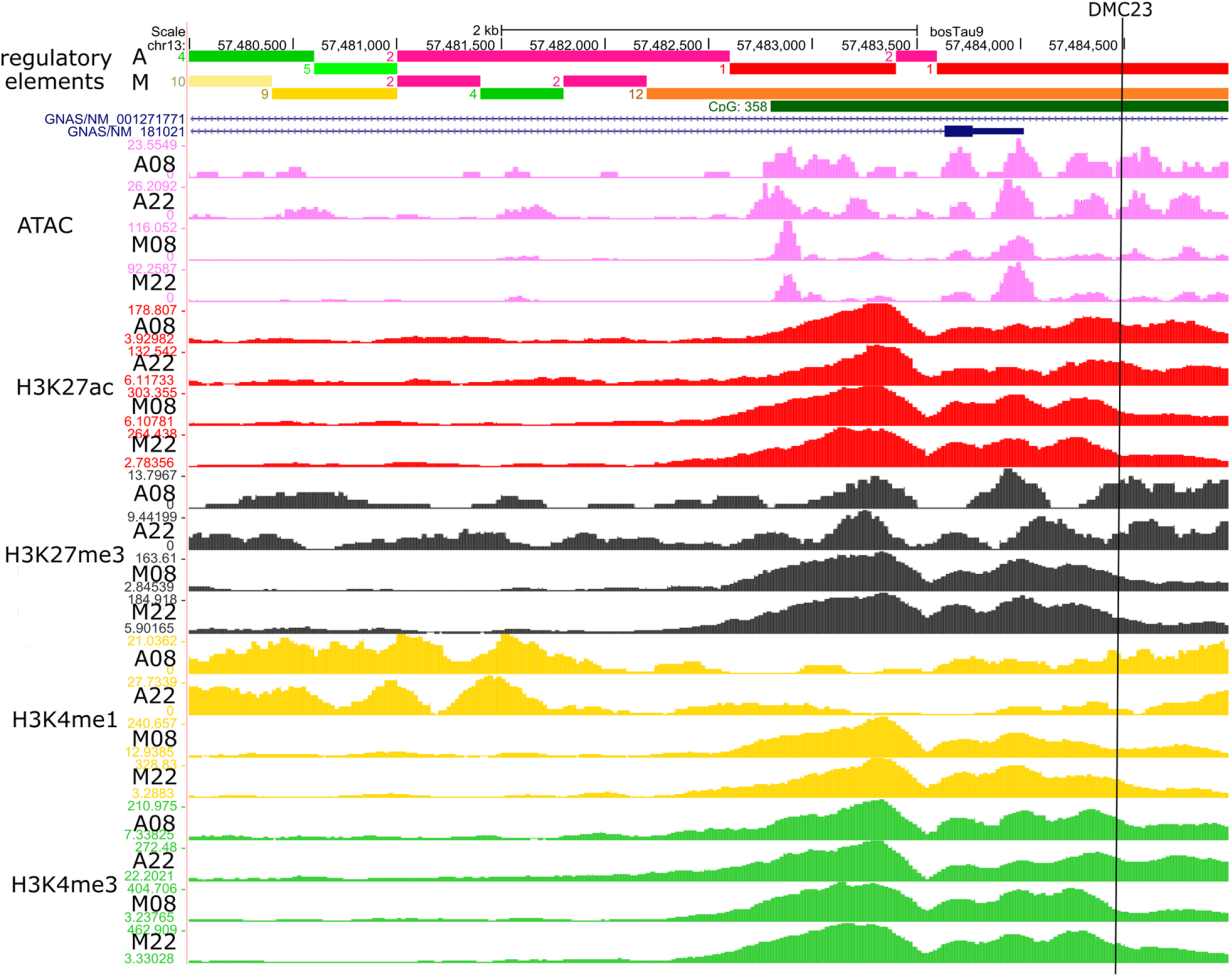
Regulatory element features of the region overlapped by the differentially methylated cytosine DMC23, which was correlated with *GNAS* expression, in adipose (A) and muscle (M). DMC23 (represented by the vertical black line) overlapped a CpG island (CpG358, represented by the dark green solid horizontal bar) in the intron 1 of *GNAS* isoform 001,271,771 and less than 1 kb from the start site of isoform 181,021. Histone marks and ATAC peaks enrichment suggested that this region was classified as bivalent/poised TSS (represented by the orange solid horizontal bar #12) in muscle (M) and strongly active promoter/transcript in adipose (A; red solid horizontal bar #1) in two male *Bos taurus* [23]. The *GNAS* gene is represented by the blue line (introns) and blue blocks (exons). Pink tracks represent the ATAC peaks. The peaks of histone marks H3K27ac, H3K27me, H3K4me1 and H3K4me3 are represented by the red, black, yellow, and green tracks, respectively. The image was obtained from UCSC Genome Browser and edited by the authors

The *GNAS* gene was predicted to be the target of two DMCs and two DMRs; however, only the DMC23, which is located in a CpG island, showed correlations between methylation and expression (Table 1, Fig. 3A). The methylation percent in the DMC23 had a high positive correlation (0.75) with *GNAS* expression (Fig. 3A). To provide further evidence that DMC23 may act as a regulatory element, we checked its position for histone modification marks and ATAC-Seq using the data provided by Kern et al. [23]. Considering that our analysis is a pool of cells taken from skeletal muscle and this may include some adipose cells that are part of the intramuscular fat, we may also have accessed the regulatory element information in adipose tissue. Figure 4 shows the enrichment of histone marks, such as H3K4me1, H3K27ac and H3K4me3, as well as an open chromatin state found by ATAC-seq in the DMC23 surrounding region (vertical black line in Fig. 4), suggesting that this region has features of an active promoter or enhancer. Indeed, in *Bos taurus* this region was classified as “strongly active promoter/TSS” in adipose tissue. However, in muscle, the presence of H3K27me3 characterizes an element repressing the expression which, together with the abovementioned epigenetic marks, may define this region as a “bivalent/poised TSS”. The importance of this region as a regulatory element was corroborated in other tissues, since all 15 tissues studied by Kern et al. [23] showed promoter features (Additional file 2: Table S10). Muscle was the only tissue which presented bivalent/poised TSS feature in this region; however, seven tissues were classified as the adipose, a.i., as strongly active promoter/TSS, two as an active TSS, while the remaining four tissues showed at gene features “promoter transcribed”.

#### *PDE4B*

Although DMC98 overlapped the gene *JAK1* and it was not correlated to this gene expression but was negatively correlated (− 0.63) with its predicted target *PDE4B* (Fig. 3B; Table 1). This gene was enriched in two pathways: adenylate cyclase-activating G protein-coupled receptor signaling pathway and voltage-gated cation channel activity. From the six selected DMCs and DMRs, this was the only one overlapping a region with no recognized regulatory element (Quiescent), showing very low signs of histone marks and ATAC in 14 tissues out of 15, according to Kern et al. [23] (Additional file 2: Table S10 and Additional file 1: Figure S7).

#### *EBF3*

**Fig. 5:**
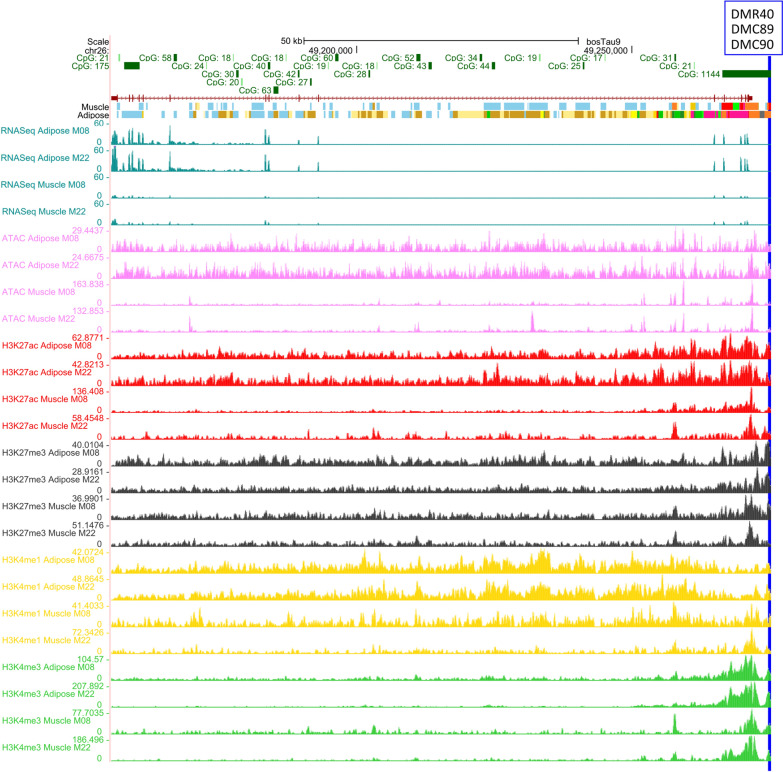
Regulatory element feature of the region overlapped by the two differentially methylated cytosines (DMC89 and DMC90) and region (DMR40) which were correlated with *EBF3* expression. DMCs and DMRs region (represented by the vertical blue line) overlapped a CpG island (CpG1144, represented by the green solid horizontal bar). The DMCs and the DMR overlap a region enriched with histone marks and ATAC peaks, which characterizes the region as strongly active promotors/transcripts in muscle and bivalent/poised TSS in adipose in two male *Bos taurus* [23]. The *EBF3* gene is represented by the dark red line (introns) and dark red blocks (exons). Pink tracks represent the ATAC peaks. The peaks of histone marks H3K27ac, H3K27me, H3K4me1 and H3K4me3 are represented by the red, black, yellow, and green tracks, respectively. The image was obtained from UCSC Genome Browser and edited by the authors

*EBF3* was the most correlated gene, having correlation with three elements (DMC89, DMC90 and DMR40) which are located in a CpG island. We found high negative pairwise correlations between *EBF3* gene expression and either DMC89 (− 0.72), DMC90 (− 0.75) or DMR40 (− 0.81) (Additional file 1: Fig. S3C–E). Other two DMCs were tested with this gene; however, no correlation was found. The *Bos taurus* data suggests that the region overlapped by the two DMCs and the DMR40 (vertical blue line in Fig. 5) has promoter features in *Bos taurus* [23] (Fig. 5, Additional file 2: Table S10). In muscle, this region was classified as strongly active promoters/transcripts which is characterized by the enrichment for two histones marks (H3K27ac and H3K4me3) and ATAC peaks. In adipose tissues, the region was part of a bivalent/poised TSS, characterized by the enrichment of all histones and ATAC marks, but mainly the H3K27me3, that has a repression function. Moreover, according to the *Bos taurus* data, *EBF3* was expressed only in adipose and muscle tissue (Additional file 1: Figure S8), with higher levels in adipose (Fig. 5).

#### *EPCAM*

The DMC12, which was selected, because it overlapped a QTL for shear force in the Nelore population, is in an intergenic region and more than 200,000 bp far away from its target *EPCAM* gene. We found a negative correlation (− 0.64) between the methylation percent of DMC12 and the *EPCAM* expression (Table 1, Fig. 3E). The region overlapped by DMC12 also showed regulatory element features in several tissues of *Bos taurus*, including adipose and muscle (Additional file 1: Figure S9). Muscle had this region classified as an ATAC island and this state seems to be conserved in other five out 15 analyzed tissues (cecum, hypothalamus, rumen, abomasum, and spleen). In adipose tissue, this region was classified as medium enhancer based on ATAC peaks (Additional file 1: Figure S9).

## Discussion

### A new layer of knowledge regarding tenderness: methylation

The genetic mechanisms underlying beef tenderness have been extensively studied in the past years; however, little is known about the epigenetic marks affecting this important production trait. Epigenetic marks, such as DNAm, may affect the phenotype without altering the genome sequence, being an additional layer of information, which may improve comprehension of the biological processes underlying beef tenderness. Furthermore, epigenetic information complements the known QTLs and genes associated with the phenotype, helping to elucidate the intermediate mechanism between genotype and phenotype. To increase the epigenetic information related to tenderness, we screened CpGs and genomic regions looking for those showing differential methylation between muscle samples with extreme beef tenderness phenotypes. We found DMCs and DMRs overlapping QTLs associated with tenderness in Nelore and overlapping regulatory elements described in *Bos taurus*, both previously identified [6, 23]. Enrichment analysis showed that signal transduction and ions channel activity may be affected by different methylation status between tender and tough beef.

### DMCs and DMRs are more highly methylated in tender than in tougher beef

The global methylation profile had low variation among animals. These findings may be, in part, due to the low genomic diversity found in the original population [25] and due to high conserved methylation profile in cattle [26]. However, we found specific CpGs and regions with differences in methylation level among the two groups. Interestingly, muscles that will become higher tenderness beefs seem to be more methylated in DMCs and DMRs than the ones that will produce tough beefs. This finding corroborates Zhao et al. [20] who found genomic regions with high or medium levels of methylation had more hypermethylated DMRs in tender than in tough Angus muscle. It thus seems that both *Bos indicus* and *Bos taurus* show the same trend of hypermethylation in tender beef. This result may be complemented by recent reports that suggests CpG methylation may be a key mechanism in the determination of myofiber type and that slow-type myofibers, which are associated with higher tenderness, had more hypermethylated promoters affecting muscle-specific gene expressions than fast-type myofibers [27]. Lu et al. [28] suggested that increasing the number of slow-type myofiber in muscle of Chinese cattle (Luxi and Quinchuan) may improve beef tenderness. Unfortunately, in the present study the type of myofibers was not characterized.

### Signal transduction and postmortem processing may be affected by methylation in muscle.

Identifying the genes that may be affected by the changes in methylation level may shed light on the mechanisms that link genotype to phenotype. In a recent study, Kern et al. [23] used several epigenetic marks and gene expression to find that most of the bovine regulatory elements do not target the nearest gene or even the gene that they overlap. These authors also reported that some regulatory elements targeted more than one gene, with an average of 1.7 genes per element in cattle. For this reason, we used their list of predicted target genes to identify those that may be affected by the DMCs and DMRs found here. Then we used the predicted target genes to perform functional enrichment analysis.

The biological processes enrichment suggests that signal transduction was affected by methylation, and this may be through the G protein signaling pathway. Four out of eleven groups of enriched pathways were directly related to G-protein-dependent signaling including adenylate cyclase-activating G protein-coupled receptor and phospholipase C-activating G protein-coupled receptor signaling pathways, regulation of Rho protein signal transduction and regulation of cytokine-mediated signaling pathway. Indeed, signal transduction through G protein-coupled is known to be a key mechanism for muscle homeostasis and growth [29]. Signal transduction mediated by G-protein is responsible for converting the extracellular stimulus, reaching the membrane in downstream cascade of processes and resulting in reactions as muscle contraction (reviewed by [30]). The hormones or other stimulus bind membrane G proteins-coupled receptor which in turn activates different subclasses of G proteins and its downstream pathways, such as adenylate cyclase-activating G protein-coupled receptor and phospholipase C-activating G protein-coupled receptor signaling pathways (reviewed by [30]), that were enriched in our analysis. Two genes grouped in the adenylate cyclase-activating pathways, *GNAS* and *PDE4B*, had their expression correlated with methylation level of the correspondent DMCs, adding further evidence to the hypothesis that G-protein signaling pathway is being affected by methylation and may affect beef tenderness. Rho protein signal transduction pathway can be activated by the G protein signaling and result in muscle contraction, this activation being mediated by Ca2+ (reviewed by [30–32]). Takano et al. [33] found Rho family G protein acting on muscle differentiation in mice. G-protein signaling pathway was found acting on skeletal muscle atrophy through cytokine-mediated signaling pathways, such as tumor necrosis factor [29, 34]. Both adenylate cyclase and phospholipase C-activating pathways regulate ion channel activities (reviewed by [30]), which was also enriched in our analysis. Specifically, our analysis indicates that calcium and potassium channels are affected by methylation which corroborates Zhao et al. [20] findings in the muscle of *Bos taurus*. The direct participation of these channels in muscle development and function is well described [35]. Tizioto et al. [6] found potassium and calcium channels enriched in a GWAS study for tenderness in a larger population of Nelore which included our animals. In addition, the enrichment of voltage-gated calcium channels and neuron-related biological processes in our analysis suggests that methylation may be affecting the muscle contraction signaling through the transfer of information from neuron to muscle in the neuromuscular junction (NMJ). The enriched calcium voltage-gated channel subunit alpha1 A (*CACNA1A*) gene encodes a subunit of the Cav2.1 channel which is located in the neuron terminal in the NMJ and has a key role in triggering muscular contraction in response to nerve impulse [36]. The increase of Ca^2+^ ions influx in the cell by the Cav2.1 stimulates the release of neurotransmitters which will hit the receptor in the muscle cell and trigger downstream reactions to muscle contraction. Conversely, *PDE4B* that was also enriched in calcium voltage-gated channels has an opposite role by restricting the amount of Ca^2+^ entering the cell via these CaV1.2 channels in cardiomyocytes [37]. Taking together these enriched pathways, we suggest that the transduction of signaling from extracellular stimulus to generation of second messengers are being affected and this may result in differences of muscle development and contraction.

Besides not related to signaling transduction, phagocytosis was one of the most associated genes enriched pathways and seems to be related with the postmortem process of the beef since muscle cells were found ingesting other cells and molecules from extracellular matrix by phagocytosis to degradation in the postmortem [38]. Moreover, Tizioto et al. [6] found phagocytosis enriched for QTLs associated with beef tenderness on day 14 after slaughter.

### DMC23 as a regulatory candidate site affecting tenderness through *GNAS* expression modulation

A key player of the G-signaling protein pathway is the G protein Gsα, encoded by the *GNAS* gene. After binding the extracellular stimulus, the membrane G protein-coupled receptors activate the Gsα which in turn stimulate the adenylyl cyclase pathway [39], enriched in our analysis. Using ATP, the enzyme adenylyl cyclase generates cyclic adenosine monophosphate (cAMP) which participates in several downstream pathways directly affecting skeletal muscle function including muscle contraction [40]. Furthermore, bovine *GNAS* is a known imprinted gene with a complex locus that has two isoforms, according to the RefSeq database. The bovine NM_001271771.1 isoform, also known as *NESP55*, has an alternative splice site on exon 2 of the *GNAS* [41, 42] and has a maternal expression in bovine fetus [41–43] and adult [41] showing a paternal methylated promoter in *B. taurus vs. B. indicus* hybrid animals [42]. The bovine NM_181021.3 isoform starts from exon 2 of *GNAS* locus and was shown to be paternally expressed in the conceptus of *B. taurus vs. B. indicus* hybrid animals [42]. We found the DMC23 and DMR20 located in the intron 1 of *GNAS* locus, overlapping the NM_001271771.1 isoform, and located less than 1 kb upstream from the TSS of the NM_181021.3 isoform. According to the pilot project from FAANG Consortium [23], these DMC23 and DMR20 overlap an active promoter region in muscle and adipose tissue and have *GNAS* as one of the target genes. It is important to keep in mind that our analysis is a pool of cells taken from skeletal muscle and this may include some adipose cells that are part of the intramuscular fat. Interestingly, the promoter was classified as poised in muscle, which is a promoter having epigenetic marks with both activation and repression effects allowing the gene to be activated or repressed as timely needed [44]. This may explain the positive correlation between DMC23 methylation and the *GNAS* gene expression found in our samples, suggesting that the methylation may have an activation effect on the expression. A hypothesis is that the methylation may be disturbing the H3K27me3 binding, decreasing the heterochromatin formation which results in higher transcription of the gene. Moreover, the status of the region overlapped by the DMC23 as an active regulatory element seems to be conserved in other tissues [23]. We present strong evidence that DMC23 overlaps a regulatory element that regulated the *GNAS* expression in muscle and adipose tissue. Therefore, DMC23 is a putative candidate site affecting tenderness due to the impact on gene expression of *GNAS,* which may affect beef tenderness through coupled receptor signaling pathways. Although little is known about the relationship of *GNAS* and tenderness, this gene was found downregulated in doubled-muscle bovine fetuses, known to result in higher shear force values [45] and enriched in the calcium signaling pathway [46]. Polymorphisms and QTLs in this gene were associated with several production traits in bovine [43, 47]. Moreover, abnormal methylation in the *GNAS* gene has been associated with disruption of several phenotypes, including diseases such as pseudohypoparathyroidism and obesity in humans [48, 49]*.*

### Differentially methylated CpG was negatively correlated with *PDE4B* expression

Meanwhile, *GNAS* gene product, Gsα, is known to activate the cAMP signaling, the phosphodiesterase enzymes (PDEs) seem to have opposite function by catalyzing the hydrolysis of cAMP decreasing its levels [50]. In skeletal muscle of rats, the PDE4 was shown to be the major cAMP hydrolyzer in comparison to the other PDEs [51]. This contradictory function of *GNAS* and *PDE4* is in agreement with our results, since we found opposite expression levels of these two genes, and these expression levels seems to be regulated by DNA methylation, providing insights on the link of methylation and beef tenderness. We found higher transcript levels of *PDE4B* in tough beef, while *GNAS* was more expressed in tender animals. This molecular pattern may suggest higher stimulation of G-protein-mediated cAMP signaling pathway in tender animals and this may reflect in the muscle functions including glycogenolysis, contractility, sarcoplasmic calcium dynamics [40] resulting in a different tenderness pattern. Interestingly, the expression of both genes was found correlated with methylation level of cytosines which were all hypermethylated in tender beef. Therefore, *GNAS* and *PDE4B* had opposite patterns of expression and methylation correlation between the beef with high and low tenderness suggesting that the signaling pathway that they partake, the cyclase-activating G protein-coupled, may have a role in the tenderness of *Bos indicus* beef and may be regulated by methylation.

### Changes in methylation level of *EBF3* promoter as a potential candidate affecting beef tenderness

The RRBS analysis identified *EBF3* as a potential gene associated with tenderness through methylation. Several pieces of evidence among our findings and previous studies assure that DMC89, DMC90 and DMR40 may have a role in regulating *EBF3* gene expression and tenderness. First, we found that these elements may regulate *EBF3* gene expression due to the high negative correlation found with methylation. This is corroborated by Kern et al. [23] that found a correlation between *EBF3* gene expression and H3K27ac signal overlapping the region, where DMC89, DMC90 and DMR40 are located. In addition, these elements are located in a promoter region and CpG island, which are known to regulate transcription and gene silencing [14]. Second, *EBF3* gene has specific functions in muscle. For instance, *EBF3* gene encodes a transcription factor that acts together with *MYOD* gene in muscle relaxation and is a regulator of muscle cell-specific transcription [52]. According to Kern et al. [23] data, *EBF3* is a tissue specific gene being expressed only in adipose and muscle, compared to other four tissues. *EBF3* is highly expressed in adipose of *Bos taurus* and with a lower expression in muscle [23]. Intramuscular fat is known to affect skeletal muscle metabolism and beef tenderness [53, 54]; therefore, *EBF3* may be acting in muscle tenderness by both muscle and adipose pathways. Taken together, these findings make DMC89, DMC90, DMR40, and the *EBF3* gene strong candidates for further studies of epigenetic events driving muscle development and tenderness.

### DMC12 overlapped QTLs for beef tenderness

Our methylation screen study resulted in regions and cytosines that are putative candidates underlying the phenotype beef tenderness in cattle, thus contributing to better understand the mechanisms regulating this trait. We highlight the DMC12 which lies within a QTL associated with tenderness in Nelore [6] and its methylation level was negatively correlated with the *EPCAM* gene expression. In *Bos taurus*, DMC12 overlapped regulatory elements in muscle and adipose [23]. The biological function of the glycoprotein *EPCAM,* a transmembrane glycoprotein mediating Ca -independent homotypic cell–cell adhesion, has been explored in cancer studies [55] and it is known to participate in cell signaling [56], but little is known about its role in cattle.

### Final considerations

Here, we adopted the RRBS approach due to its advantages as base-level resolution and the accessible cost. However, this approach limited the analysis to part of the genome, which might contribute to the small variation in methylation profile found between the groups when compared to another study in Angus [20]. Despite this limitation, we were able to provide new molecular insights to beef tenderness, which is a complex trait with a lot of its underlying mechanisms still undeciphered. We identified and suggested candidate regions and CpGs that may shed light on epigenetic mechanisms modulating beef tenderness by screening methylation on the *Bos indicus* muscle genome and taking advantage of previous information about regulatory elements and QTLs. Our results increase previous knowledge about mechanisms affecting beef tenderness in *Bos indicus*, since as far as we know, there are no previous studies in this subspecies. Further studies are needed to answer complementary questions, among them, whether these methylation patterns are the cause or consequence of gene expression and beef tenderness, whether it was affected by the environment and how much it is inherited across generations.

## Conclusions

We identified changes in DNAm level associated with tenderness, and functional analysis suggests that this link may be through modulating signal transduction, more specifically the G-protein-dependent signaling pathways, that have a specific function in muscle homeostasis. We also found strong evidence that differences in methylation level associated with tenderness are modulating *GNAS* and *EBF3* gene expressions, thus making these genes potential candidates associated with the phenotype. The FAANG pilot project data in *Bos taurus* helped to identify potential functional role of the DMCs and DMR overlapping. Further studies of the DMCs and DMRs overlapping QTLs for SF may help complement the knowledge of the epigenetic mechanisms participating in the makeup of beef tenderness.

## Methods

### Experimental design and animals

This study aimed to identify epigenetics marks that may contribute to muscle development and resulting in differences in beef tenderness of *Bos indicus*. For this we investigated the association of DNA methylation profile in Nelore (*Bos indicus*) skeletal muscle by comparing contrasting animals for tenderness. The study population (*n* = 200) was composed of Nelore steers raised and kept on feedlots at Embrapa Southeast Livestock in São Carlos-SP, Brazil, as described by Tizioto et al. [57]. The steers were sired by 33 registered Nelore bulls belonging to the main family lineages that make up the Nelore breed in Brazil. Steers were raised in feedlots with identical nutritional rations and handling conditions until slaughter at an average age of 25 months.

### Phenotypes and sample selection.

Beef tenderness measurements were described by Tizioto et al. [6]. Muscle samples from the *Longissimus thoracis* skeletal muscle located between the 12th and 13th ribs were collected 24 h after slaughter. Steaks were used to measure the SF using a TA XT2i (Stable Micro Systems Ltd., Surrey, United Kingdom) texture analyzer coupled to a Warner–Bratzler blade, at 1.016 mm thickness [58]. Samples were kept in a 2 °C chamber for aging and measurements of SF were obtained at 14 days after slaughter. Muscle samples were selected from the initial population by ranking the estimated breeding value (EBV) for SF (EBVSF) of each animal. The EBVs were estimated using standard best linear unbiased prediction (BLUP) procedures under an animal model performed using the mixed procedure of SAS [59–61] as reported by Gonçalves et al. [8]. Twelve samples were selected and divided into two groups with extreme values and were named according to the level of tenderness: highest values of EBVSF (*n* = 6), corresponding to the lowest tenderness, were named as “Tough”, while the lowest values of EBVSF (*n* = 6), corresponding to the highest tenderness, were named as “Tender”. Only animals born from different sires were considered for each group to avoid the confounding between phenotype and infinitesimal effect of sire.

### DNA and RNA extraction and quality evaluation

*Longissimus thoracis* skeletal muscle samples were collected at slaughter, stored in liquid nitrogen, and kept in a freezer at − 80 °C until processing. DNA extraction was carried out using the DNeasy*®* Blood & Tissue kit (Qiagen Inc., Germantown, MD, USA), according to the manufacturer's protocol. DNA concentration was measured using Qubit dsDNA High Sensitivity Assa*y* (Thermo Fisher Scientific Waltham, MS, EUA) and the quality measured by Fragment Analyzer*™* and the DNF-487 Standard Sensitivity or the DNF-488 High Sensitivity genomic DNA Analysis *Kit* (Advanced Analytical).

### Reduced representation bisulfite sequencing (RRBS) and processing

The RRBS experiments were performed by Diagenode® (Seraing, Belgium). Each sample library was prepared from 100 ng of genomic DNA using the Premium RRBS kit (Diagenode), according to the manufacturer's protocol. Bisulfite conversion efficiency independent of CpG context was assessed by adding methylated and unmethylated spike-in controls (0.1% concentration). Briefly, the protocol consists in the digestion of DNA by *MspI* enzyme followed by the fragment end repair, and addition of adaptors. Next, samples were quantified by qPCR and the Ct values were used to pool samples by similarity. Bisulfite conversions were performed using the Premium RRBS kit (Diagenode) followed by library enrichment by PCR, based on the manufacturer’s protocol. Adequate fragment size distributions were confirmed by Bioanalyzer High Sensitivity DNA chips (Agilent). Libraries were sequenced on Illumina HiSeq 3000 using single-end 50 bp reads. Sequencing read quality control was performed using FastQC version 0.10.1(https://www.bioinformatics.babraham.ac.uk/projects/fastqc/) and adaptors were removed by Trim Galore! version 0.4.1. (http://www.bioinformatics.babraham.ac.uk/projects/trim_galore/). Bismark version 0.23.0 [62] was used to align the reads to the *Bos taurus* reference genome UCD1.2/bosTau9 and to identify methylated cytosines. The reference genome UCD1.2/bosTau9 fasta file was downloaded from UCSC Genome Browser in February/2021(http://hgdownload.soe.ucsc.edu/goldenPath/bosTau9/bigZips/bosTau9.fa.gz).

### RNA sequencing and processing

Eleven animals, out of the 12 used to analyze for DNA methylation, also had RNA sequencing data. These 11 RNA samples were a subset of the 200 reported by Diniz et al. [63]. The methods for sample collection, total RNA extraction, library preparation, sequencing and read processing are described by Diniz et al. [63]. In summary, muscle samples were collected immediately after slaughter, snap frozen in liquid nitrogen and kept at − 80 °C until RNA extraction. From 100 mg of frozen tissue, total RNA was isolated using Trizol® standard protocol (Life Technologies, Carlsbad, CA, United States) and the resulting mRNA was evaluated in the Bioanalyzer 2100® (Agilent, Santa Clara, CA, United States). The cDNA libraries were generated using Illumina TruSeq® RNA Sample Preparation Kit v2 (San Diego, CA, United States), followed by purification and validation using Agilent 2100 Bioanalyzer (Santa Clara, CA, United States). Library preparation and sequencing were conducted by ESALQ Genomics Center (Piracicaba, SP Brazil). Paired-end (PE) sequencing was executed on Illumina Hiseq 2500® (San Diego, CA, United States) platform following standard protocols. Read processing protocol consisted in filtration using Seqyclean package version 1.4.13 [64], and quality control using FastQC version 0.11.2 [65] and MultiQC version 1.4 [66].

### Differential DNA methylation analysis between extremes

The comparison between the data set from two extreme groups for EBVSF phenotypes was executed using methylKit version 1.8.1 [24] in R version 3.5.1 [67]. First, all cytosines were filtered for minimum coverage of 10 reads and maximum percentile of 99.9%. The methylKit function that normalize the coverage was applied using default settings. A Chisq-test was used to define the cytosines or genomic regions that were differentially methylated (DMCs or DMRs, respectively) between the two groups of animals. A correction for overdispersion was applied, as suggested by methylKit user guide. The DMRs window size was 100 bp and both DMCs and DMRs were filtered for at least 25% of difference of methylation between groups and *q*-value ≤ 0.05.

### Gene annotation and functional enrichment analysis

Gene annotation was carried out using the package Genomation version 3.8 [68] through Methylkit version 1.8.1 [24] in R. The NCBI RefSeq annotation bed file used was obtained from UCSC Genome Browser. The webtool Faangmine (http://128.206.116.18:8080/faangmine/) was also used to find genes overlapping the DMCs and DMRs. Promoters were considered as 1000 bp upstream and downstream from the gene’s TSS, following the default of Methylkit. CpG island and shore regions’ information were obtained from UCSC Genome Browser [69]. To retrieve information whether DMCs and DMRs were overlapping regulatory elements, we used the FAANG pilot project [23]. These data provided information about the regulatory element status of the region based on enrichment of histone marks (H3K27me3, H3K4me1, H3K27ac, H3K4me3), ATAC-Seq and CTCF data in 15 tissues of two male *Bos taurus*. The method to classify the genomic region regarding the regulatory element state was described by Pan et al. [70]. In summary, the epigenetic marks data described above were used to train a chromatin state prediction model in ChromHMM69 (v.1.20) and 15 states were chosen. We also used these data to find the target genes of the DM regions and the parameters used to download this information from UCSC were Assembly *Apr. 2018 (ARS-UCD1.2/bosTau9)*, group *UC Davis FAANG Pilot Project* and track *Cattle Regulator-Gene Interactions* to retrieve the target genes. The UCSC Table Browser tool [69] was accessed in May of 2021. Among 15 tissues available in this database, we focused our analysis on muscle but, in a lesser extent, on adipose tissue as well, since our samples of skeletal muscle may contain some adipose cells that are part of the intramuscular fat. The list of predicted target genes was submitted to functional enrichment analysis using ClueGO v. 2.5.5. software and CluePedia v. 1.5.5 [71]. Significantly enriched GO and BP terms were declared with a right-sided hypergeometric test at a Benjamini and Hochberg adjusted *p* < 0.05 [72]. We searched for DMCs and DMRs whose position overlapped QTLs previously associated with tenderness by GWAS [6] in the original population. QTL positions obtained from Tizioto et al. [6] using the UMD 3.1.1 reference genome were converted for the ARS-UCD1.2 version using Lift Genome Annotations UCSC tool (https://genome.ucsc.edu/cgi-bin/hgLiftOver). We also used the webtool Faangmine (http://128.206.116.18:8080/faangmine/) to find tenderness QTLs from other populations in the CattleQTLdb [73], and overlapped the positions with the DMCs and DMRs genomic positions.

### Development of a manually curated list of candidate methylation regions and cytosines affecting tenderness

We used the initial genome-wide DNAm screen together with information available in the literature to create a manually selected list of regions or CpGs that are potentially acting as regulators of tenderness and whose differences in methylation level may lead to phenotypic changes. First, we selected all the DMCs and DMRs that overlapped QTLs for shear force. We included the target genes and the closest gene of these DMCs and DMR. Then, all target genes that were clustered in any of the enriched gene ontology biological processes were included to the list. Finally, we complemented the list with DMCs/DMRs whose target genes had function related with muscle development or tenderness, based on literature information. The manually curated list of candidate DMCs/DMRs and genes were submitted to statistical association tests between methylation and expression of the target genes.

### Correlation between methylation percentage and gene expression

To investigate the relationship between DMCs or DMRs and its respective target genes, we carried out statistical associations between methylation level and gene expression. The methylation percentage of DMCs and DMRs were obtained from methylKit version 1.8.1 [24] in R using the function percMethylation(). To access the gene expression of target genes, the pre-processed reads obtained from RNA-Seq were used. High-quality reads were filtered to remove extremely low expressed genes among the 11 samples, retaining genes with at least seven non-zero samples. Count normalization was executed using the standard method from DESeq2 version 1.28.1 [74]. The correlation between the DMC or DMR percentage of methylation and read counts was analyzed using Pearson correlation in R by cor.test() function. For those genes whose expression were correlated to methylation, we also tested whether the expression was different between the groups of extreme phenotypes. For this we used Wilcoxon test applying the R function wilcox_test() with paired = False.

## Supplementary Information

**Additional file 1.** Supplementary Figures S1–S9.

**Additional file 2.** Supplementary Tables S1–S10.

## Data Availability

The data set generated and analyzed during the current study regarding the methylation experiments is available in the NCBI’s Gene Expression Omnibus (GEO) under accession numbers GSE190966. The RNA-Seq data set is available in the European Nucleotide Archive (ENA) repository (EMBL-EBI), under accession PRJEB13188, PRJEB10898, and PRJEB19421 (https://www.ebi.ac.uk/ena/submit/sra/). The data set regarding the histone marks, ATAC-Seq and regulatory elements are available at http://farm.cse.ucdavis.edu/~ckern/Nature_Communications_2020/.

## Abbreviations

cAMP: Cyclic adenosine monophosphate
CpG: Cytosine followed by guanine in a linear sequence
CpGi: CpG island
DMC: Differentially methylated cytosines
DMR: Differentially methylated regions
DNAm: DNA methylation
*EBF3*: EBF transcription factor 3
EBVSF: Estimated breeding value for shear force
*EPCAM*: Epithelial cell adhesion molecule
*GNAS*: GNAS complex locus
GO: Gene ontology
NMJ: Neuromuscular junction
*PDE4B*: Phosphodiesterase 4B
QTL: Quantitative trait loci
RRBS: Reduced Representation Bisulfite Sequencing
SF: Shear force
