# Supplementary material for: DNA methylation may affect beef tenderness through signal transduction in Bos indicus
Source: Epigenetics Chromatin. 2022 May 13;15:15. doi: 10.1186/s13072-022-00449-4 (PMC9107245; doi:10.1186/s13072-022-00449-4)
Supplement: Supplementary file 1 — Additional file 1. Supplementary Figures S1–S9. [file 13072_2022_449_MOESM1_ESM.docx]

**DNA methylation may affect beef tenderness through signal transduction in *Bos indicus***

Marcela Maria de Souza^1,2^, Simone Cristina Méo Niciura^1^, Marina Ibelli Pereira Rocha^1,3^, Zhangyuan Pan^4^, Huaijun Zhou^4^, Jennifer Jessica Bruscadin^1,3^, Wellison Jarles da Silva Diniz^1,5^, Juliana Afonso^1^, Priscila Silva Neubern de Oliveira^3^, Gerson B. Mourão^6^, Adhemar Zerlotini^7^, Luiz Lehmann Coutinho^6^, James E. Koltes^2^, Luciana Correia de Almeida Regitano^1*^

^1^Embrapa Pecuária Sudeste, Empresa Brasileira de Pesquisa Agropecuária, São Carlos, Brazil

^2^Department of Animal Science, Iowa State University, Ames, United States

^3^Department of Genetics and Evolution, Federal University of São Carlos, São Carlos, Brazil

^4^Department of animal Science, University of California, Davis, California, United States

^5^Department of Animal Science, Auburn University; Auburn, Alabama, United States

^6^Department of Animal Science, Luiz de Queiroz College of Agriculture, University of São Paulo, Piracicaba, Brazil

^7^Embrapa Informática Agropecuária, Empresa Brasileira de Pesquisa Agropecuária, Campinas, Brazil

Correspondence: [luciana.regitano@embrapa.br](mailto:luciana.regitano@embrapa.br)

**Additional file 1: Supplementary Figures**


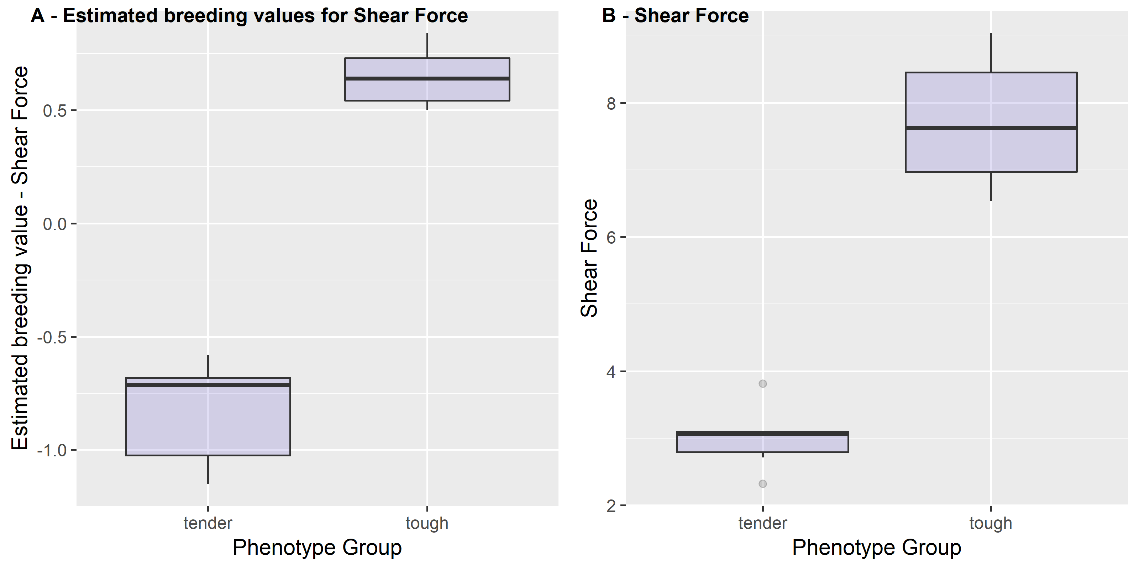


**Figure S1.** Box plot showing the distribution of shear force: **(A)** estimated breeding values for Shear Force and **(B)** Shear Force values.


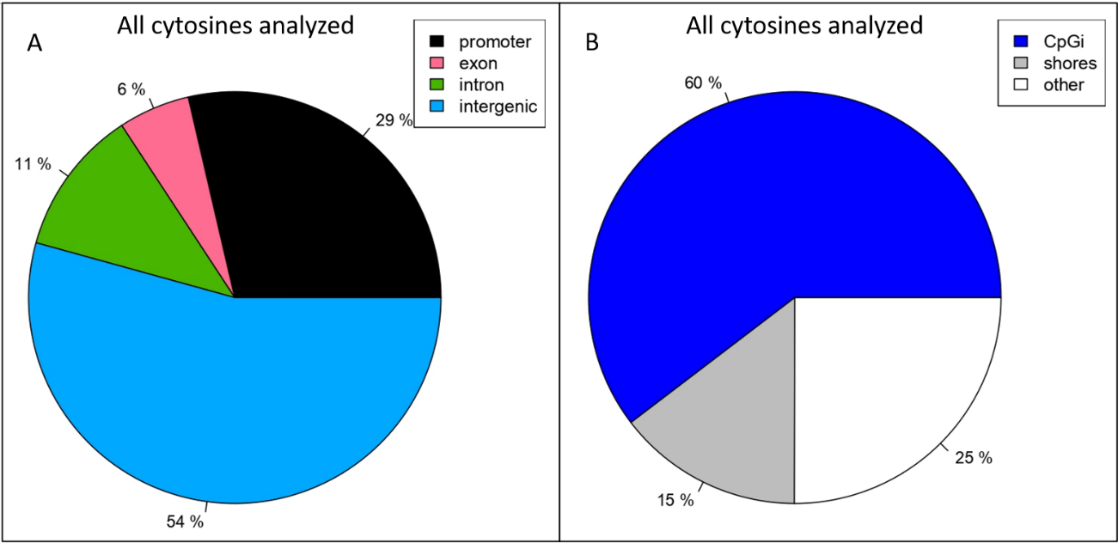


**Figure S2.** Locations of all CpGs used for differential methylation analysis (n=635469). **A.** Percent of CpGs in promoters, gene body (exons and introns) and intergenic regions. **B.** Percent of CpGs overlapping CpG islands (CpGi) and shore CpGs.


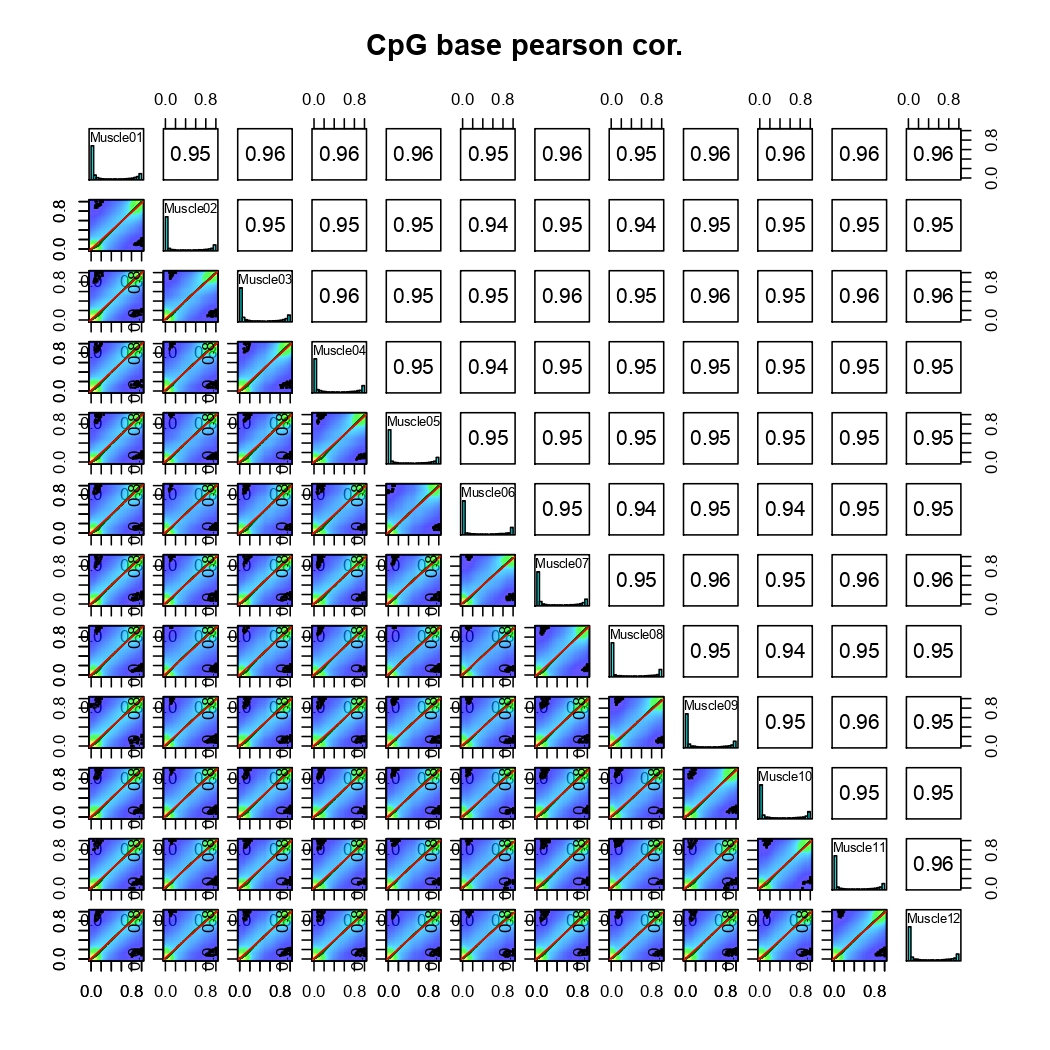


**Figure S3.** Sample correlation. Sample Correlation. Scatter plot and correlation coefficients among all samples.


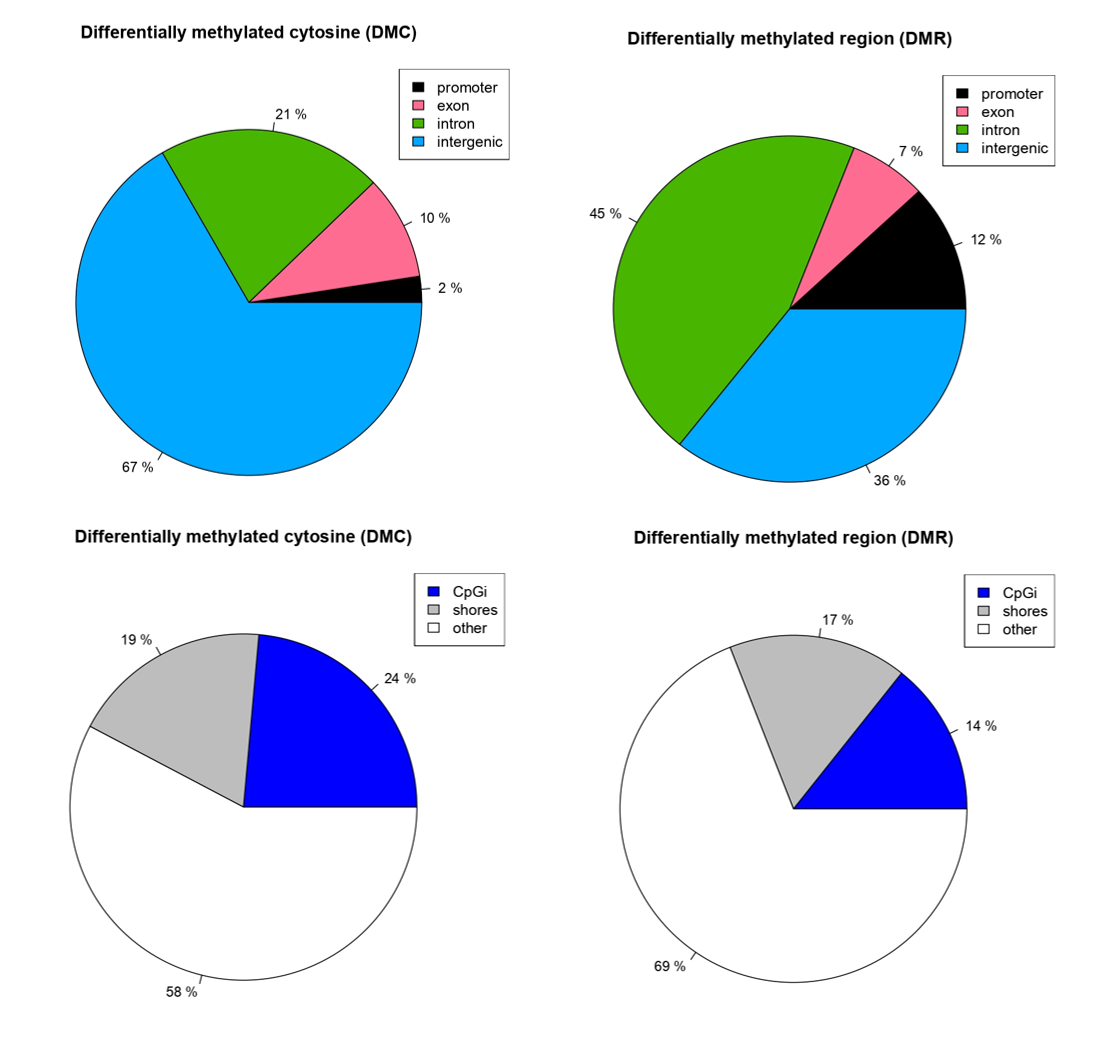


**Figure S4**. Proportion of differentially methylated cytosines (DMCs) (**A**) and differentially methylated regions (DMRs) (**B)** overlapping promoter, exon, intron or intergenic regions. **C** and **D** shows the proportion of DMCs and DMRs, respectively, overlapping CpG islands (CpGi), shore regions or other.


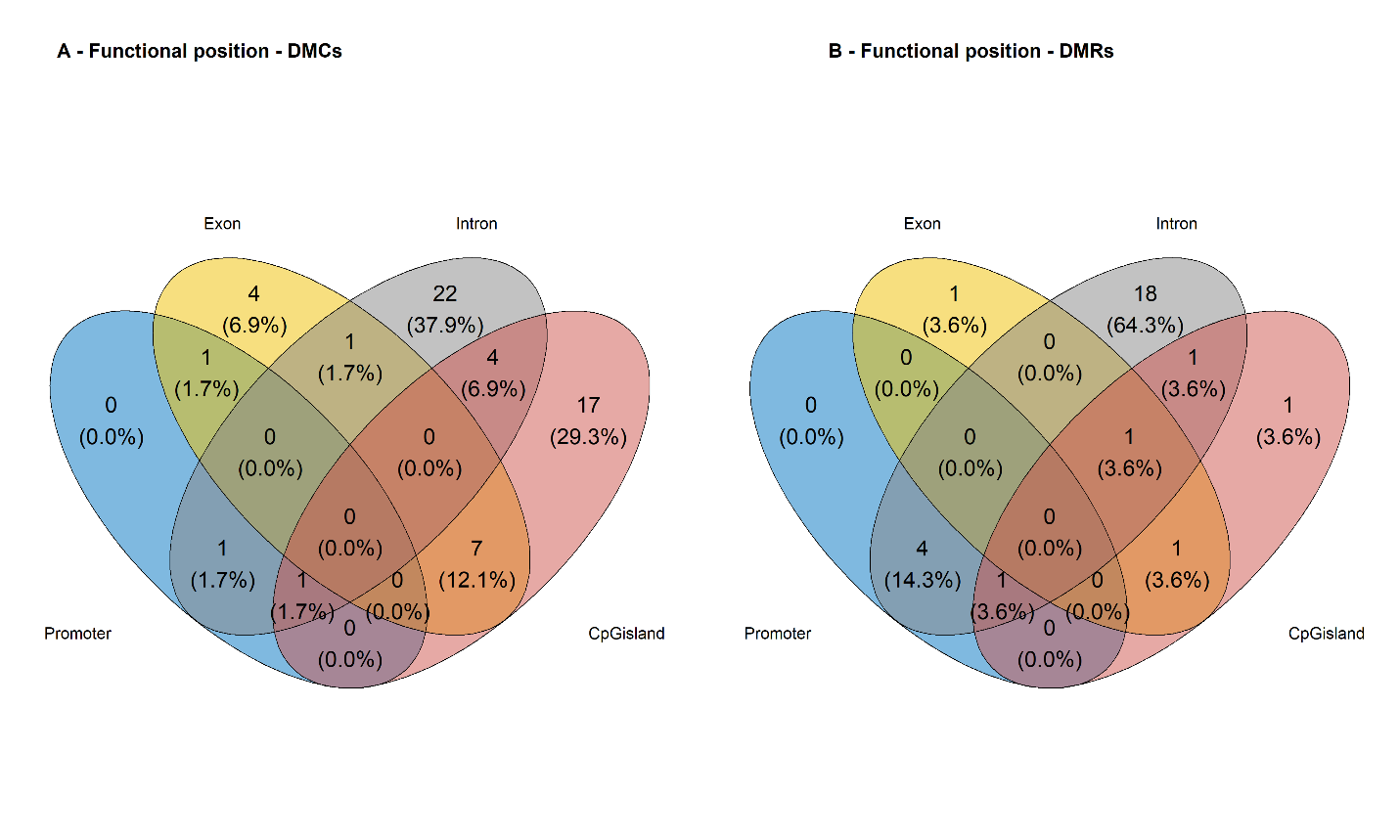


**Figure S5.** Venn Diagram showing the distribution of differentially methylated cytosines (DMCs) (A) and differentially methylated regions (DMRs) (B) overlapping promoters, exons, introns and CpG islands. This figure shows that some DMC and DMR were located in more than one functional location.


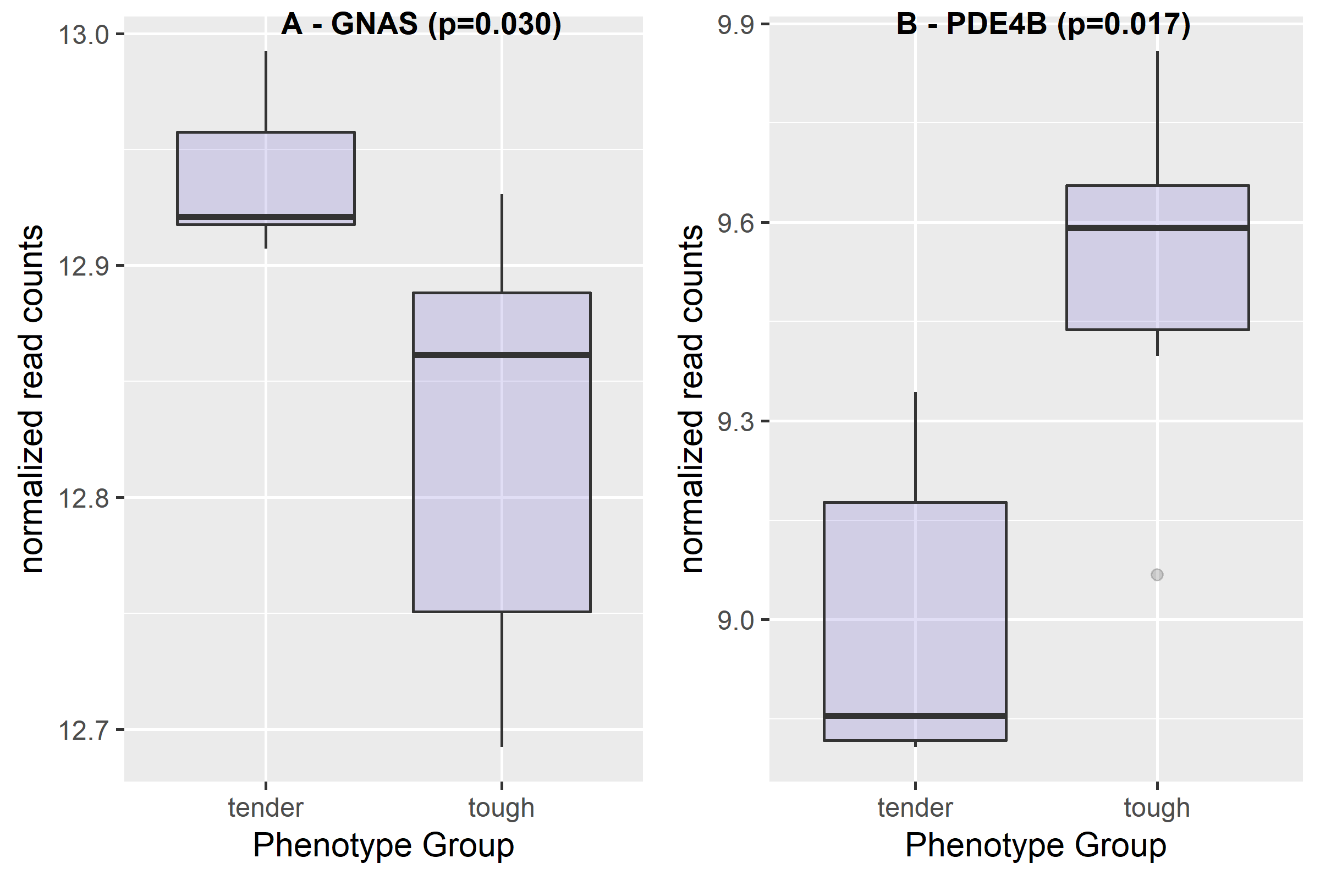


**Figure S6.** Box plot showing the expression (normalized counts) distribution of (A) GNAS gene and (B) PDE4B between the two extremes phenotypes of shear force (tender = lowest shear force, tough = highest shear force breeding values).


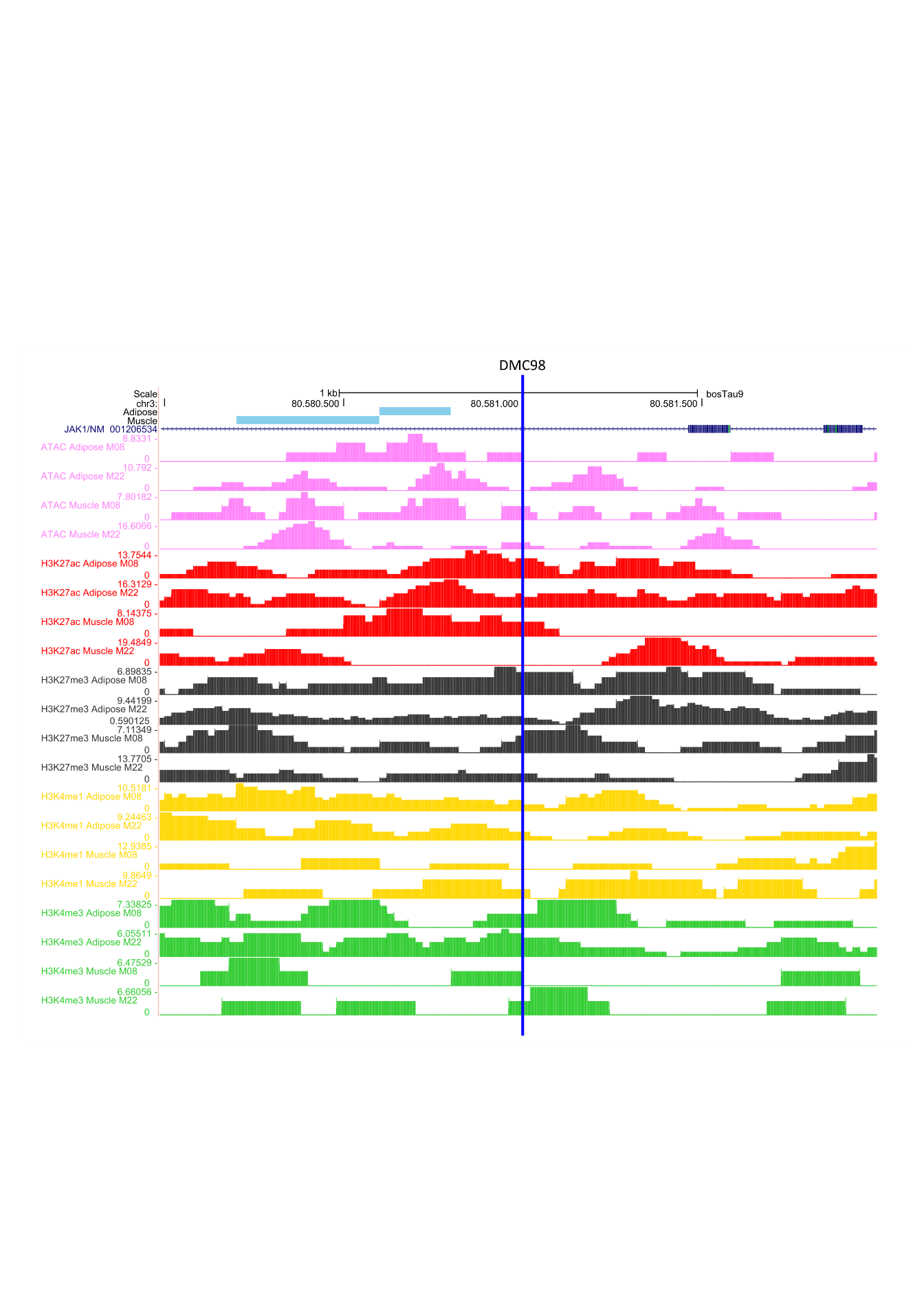


**Figure S7.** Regulatory element features of the region overlapped by the differentially methylated cytosines DMC98, which was correlated with PDE4B expression. DMC98 is located (represented by the vertical blue line) in an intron of JAK1 gene. Low enrichment of histone marks and ATAC peaks suggested that this region was classified as quiescente (#15) in muscle (M) and in adipose in two male Bos taurus (Kern et al., 2021). Pink tracks represent the ATAC peaks. The peaks of histone marks H3K27ac, H3K27me, H3K4me1 and H3K4me3 are represented by the red, black, yellow, and green tracks, respectively. The image was obtained from UCSC Genome Browser and edited by the present authors.


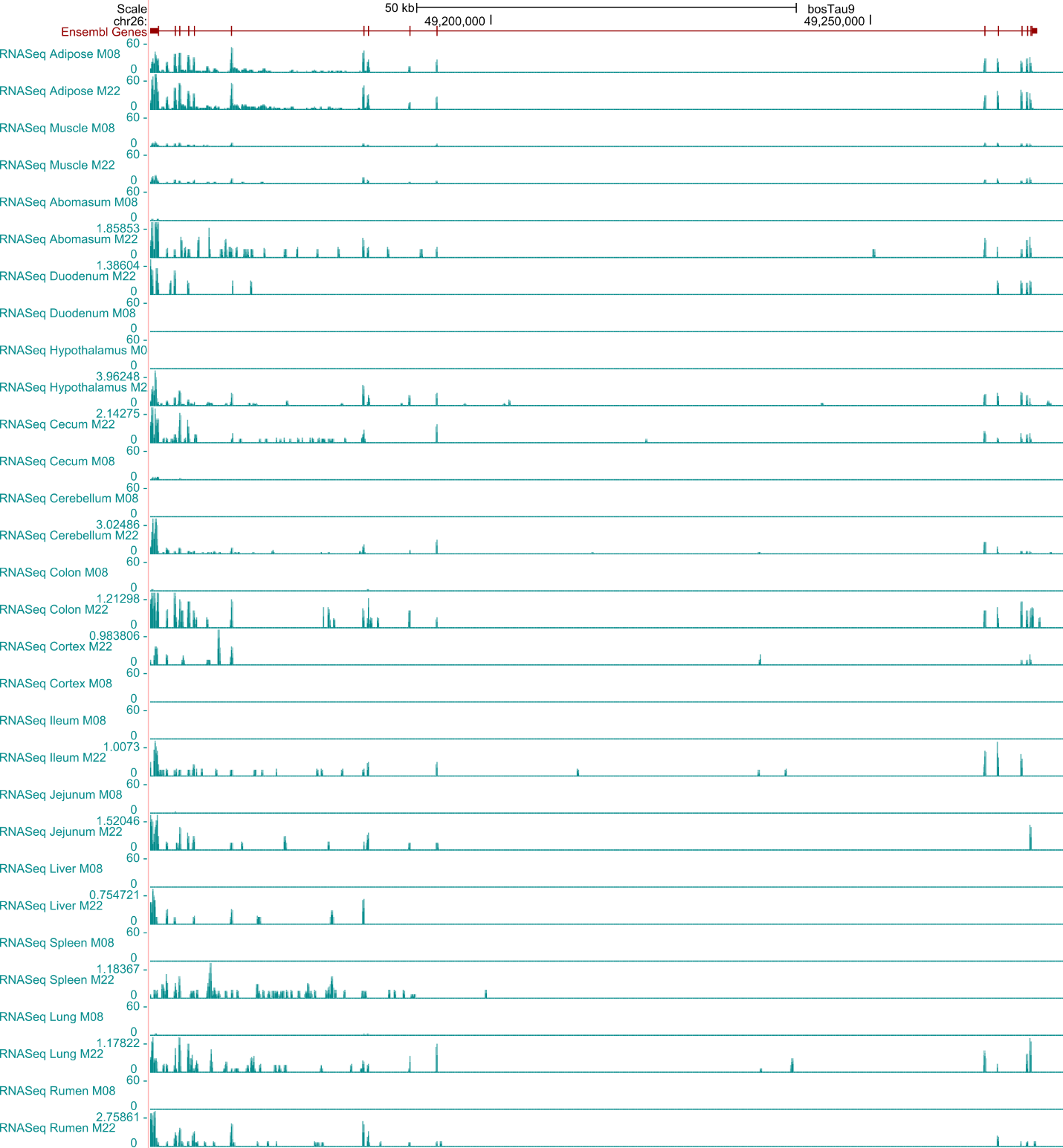


**Figure S8.** EBF3 Expression using RNA-Seq data of 15 tissues obtained from two male Bos taurus. Data available in Kern et al., 2021.


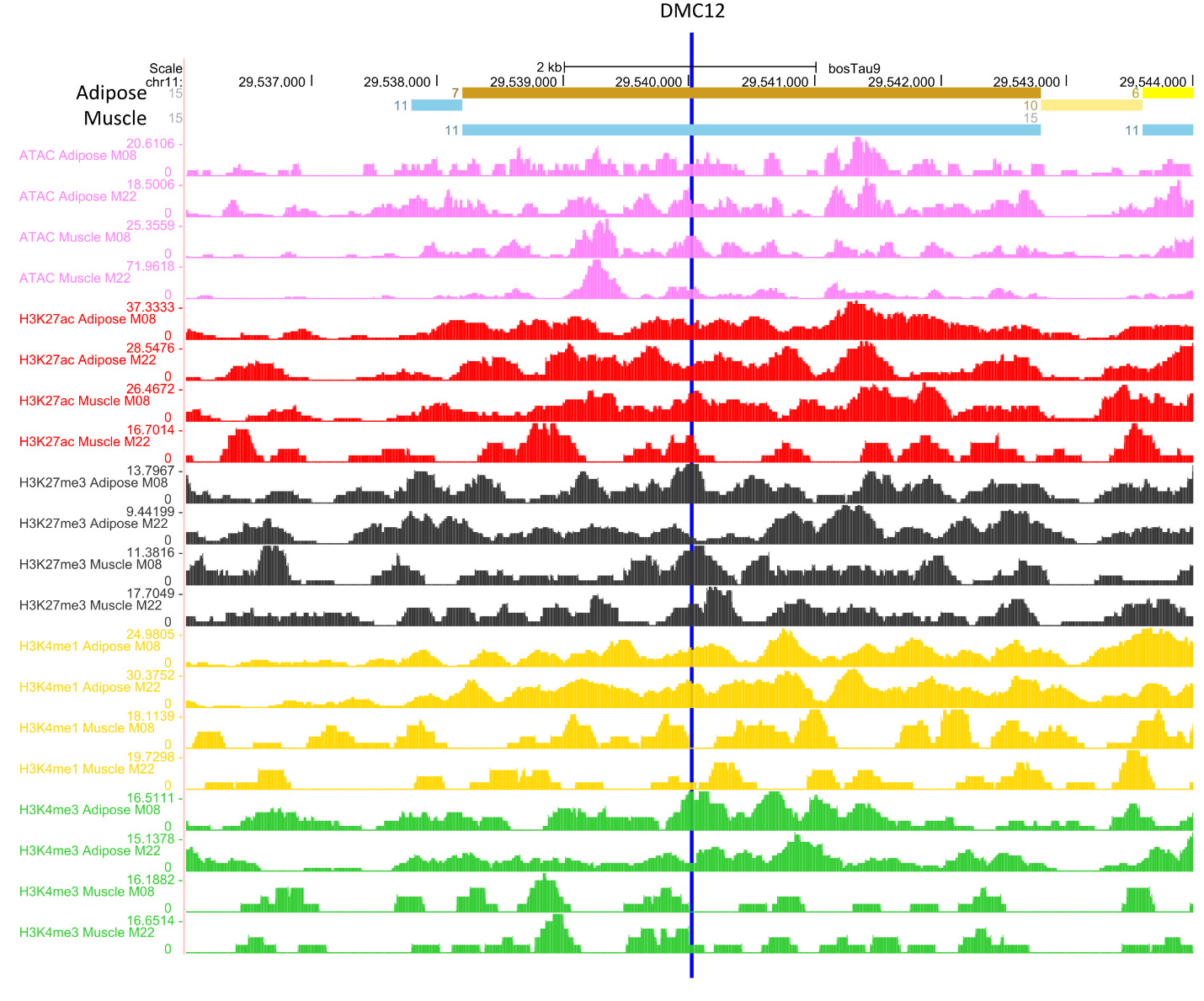


**Figure S9.** Regulatory element features of the region overlapped by the differentially methylated cytosines DMC12, which was correlated with EPCAM expression. DMC12 (represented by the vertical blue line) in an intergenic region and more than 200,000 bp far away from its target EPCAM. Histone marks and ATAC peaks enrichment suggested that this region was classified as ATAC island (represented by the light blue solid horizontal bar #11) in muscle (M) and medium enhancer with ATAC in adipose (A; dark yellow solid horizontal bar #7) in two male Bos taurus (Kern et al., 2021). Pink tracks represent the ATAC peaks. The peaks of histone marks H3K27ac, H3K27me, H3K4me1 and H3K4me3 are represented by the red, black, yellow, and green tracks, respectively. The image was obtained from UCSC Genome Browser and edited by the present authors.
